# Preprints in medical research: Progress and principles

**DOI:** 10.1371/journal.pmed.1002563

**Published:** 2018-04-16

**Authors:** Larry Peiperl, on behalf of the PLOS Medicine Editors

**Affiliations:** Public Library of Science, San Francisco, California, United States of America and Cambridge, United Kingdom

## Abstract

In this month’s editorial, the *PLOS Medicine* Editors discuss the role of preprints in human health research and propose a 3-part framework for ensuring benefit.

Sharing of preprints—scientific manuscripts that are posted in a publicly accessible, online repository before peer review for journal publication—can accelerate access to information in infectious disease outbreaks, according to an article published earlier this month by Michael Johansson and colleagues in *PLOS Medicine*. In an analysis of published articles and those posted on preprint servers, the authors found that preprint posting increased during the 2015–17 outbreak of Zika in the Americas relative to the 2014–15 Ebola outbreak in West Africa, and that preprints, while still used for only a small proportion of papers, provided much earlier access to scientific findings [1].

For medical journal editors who agreed in late 2015 and early 2016 that authors should not be penalized for sharing results in public health emergencies [2–4], these results signal gratifying progress over practices in 2003, when the severe acute respiratory syndrome (SARS) epidemic was shown to have ended before 93% of journal articles on the epidemiology of SARS were published [5].

*PLOS Medicine* has long permitted the posting of preprints for research articles submitted to the journal, and this remains our policy. For the most part, posting of preprints has been occasional, undertaken on the initiative of authors without the journal’s involvement. In the coming months, PLOS journals that handle research in more basic biological fields will begin facilitating preprint posts of journal submissions, with author approval, on the bioRxiv preprint server operated by Cold Spring Harbor Laboratory [6]. *PLOS Medicine* will not initially be offering transfer of submitted manuscripts to bioRxiv as preprints. Because the journal’s scope encompasses clinical and policy-related research, we believe it is important now to establish appropriate standards for the early sharing of research that has potential implications for human health but has not been peer reviewed. As preprint servers for clinically focused research become more widely available and preprint posting becomes more routine and more closely integrated with journal publishing, editors of medical journals should join the conversation about best practices for preprint sharing in medical research.

In calling for wider use of preprints, Johansson and colleagues note the importance of protecting participant confidentiality, ensuring that posted research was ethically conducted, and guarding against misinterpretation of results that have yet to undergo peer review, either by traditional journal routes or through newer, more public venues [1]. In medical research, competing interests have been shown to be associated with reported outcomes [7], and editors and peer reviewers persuade authors to tone down overstated conclusions prior to publication as a matter of course. At the same time, the press and other media networks are expected to disseminate the latest available information, and even readers with an urgent interest in a given health-related topic may be hard pressed to assess the validity of research methodology. As publication practices evolve, what is the best way for the medical research community to fulfill the Hippocratic imperative to do no harm while avoiding undue delays that may result in harm?

A constructive solution will reflect the legitimate priorities—and depend on the engagement—of several groups. We propose a framework of three broad categories: transparency in reporting; clarity about the role of a preprint versus a peer-reviewed journal article; and responsibility for safety.

First, preprints of relevance to human health should incorporate the best practices that medical journals have developed for transparency in reporting. Funding sources, author competing interests, and involvement of funders in creating or deciding to post the preprint should be declared. For clinical trials, preprints should include reference to the trial’s registration in a WHO-approved database [8] and should follow the widely accepted CONSORT reporting guideline for clinical trials [9]. For observational and epidemiological studies, preprints should indicate whether analyses were prospectively determined (in which case the prespecified analysis plan should be included) or exploratory in nature. Ensuring that practices to promote transparency, which should already be part of manuscript preparation, translate to preprints will support their legitimacy and usefulness as a form of scientific communication.

Second, participants in all stages of the information sharing process should maintain the distinction between a preprint and a peer-reviewed article. For example:

- Preprints should be clearly marked (for example, with a watermark), in all accessible forms (online, pdf, etc.) as not peer reviewed nor accepted for journal publication. Reporters and media outlets that cover health research should be informed by research and publishing communities, and in turn should undertake to educate the public, that preprints are intended for researchers familiar with limitations of the study methodology, and that results or conclusions may change during peer review, which often includes assessment of statistical methodology.
- Authors and institutions should not seek media attention for preprints, and if contacted by the press should acknowledge that the content has not been peer reviewed. If the preprint does receive press coverage, authors should later apprise reporters of any changes between preprint and published article that substantially alter clinical or policy implications, and should understand that journals may also alert the media to such changes.
- Preprint servers should indicate when journal publication has occurred and provide a link from the preprint to the published article. Journal articles preceded by a preprint should include a reference to the preprint. Journal editors may ask authors to summarize substantive differences between the preprint and peer-reviewed version of their work.

These practices will clarify relationships between preprint and published versions and discourage unwarranted or uncritical application of information contained in preprints.

Third, preprints should not be posted when the reported information could be misapplied to pose a significant threat with potential consequences to health and safety. No single list or algorithm can identify all situations in which potential errors or misinterpretations are likely to cause harm that could be avoided by not posting. Sound judgment by authors, journal editors (when involved in screening or facilitating preprint posts), and preprint services must inform the decision to post a preprint. For some manuscripts, timely expert review before public sharing may remain the optimal approach. In all cases, preprints must adhere to established standards for the ethical conduct of human research, including the protection of participant confidentiality.

These points do not constitute journal policy, and cannot; their implementation goes beyond the remit of journal editors to involve the broader community of journalists, preprint service providers, institutions and researchers themselves. Nor are these points exhaustive; as in other areas of research it will also be important to address such practical issues as copyright licensing for preprints and community standards around “scooping” when a journal submission is antedated by a preprint on a similar topic by different authors.

As opportunities for preprint sharing advance, medical journals should endeavor to ensure that the resulting changes benefit human health and support the public understanding of medical research. We hope these ideas will promote wider discussion among researchers and research participants, preprint posting services, journalists and journal editors. We welcome your comments.
